# Screening of TNFR1 Binding Peptides from *Deinagkistrodon acutus* Venom through Phage Display

**DOI:** 10.3390/toxins14020155

**Published:** 2022-02-19

**Authors:** Kangran Zhang, Yang Liu, Yezhong Tang

**Affiliations:** 1 Chengdu Institute of Biology, Chinese Academy of Sciences, Chengdu 610041, China; zhangkangran18@mails.ucas.ac.cn; 2 Savaid Medical School, University of Chinese Academy of Sciences, Beijing 100049, China

**Keywords:** snake venom, phage display, surface plasmon resonance (SPR), tumor necrosis factor (TNF), peptide interaction, *Deinagkistrodon acutus*

## Abstract

The venomous species *Deinagkistrodon acutus* has been used as anti-inflammatory medicine in China for a long time. It has been proven to have anti-inflammatory activity, but its specific anti-inflammatory components have not yet been fully elucidated. Tumor necrosis factor receptor-1 (TNFR1), which participates in important intracellular signaling pathways, mediates apoptosis, and functions as a regulator of inflammation, is often used as the target to develop anti-inflammatory drugs. The small peptides of snake venom have the advantages of weak immunogenicity and strong activity. To obtain the specific TNFR1 binding peptides, we constructed a T7 phage library of *D. acutus* venom glands, and then performed biopanning against TNFR1 on the constructed library. After biopanning three times, several sequences with potential binding capacity were obtained and one 41-amino acid peptide was selected through a series of biological analyses including sequence length, solubility, and simulated affinity, named DAvp-1. After synthesis, the binding capacity of DAvp-1 and TNFR1 was verified using surface plasmon resonance technology (SPR). Conclusively, by applying phage display technology, this work depicts the successful screening of a promising peptide DAvp-1 from *D. acutus* venom that binds to TNFR1. Additionally, our study emphasizes the usefulness of phage display technology for studies on screening natural product components.

## 1. Introduction

Snake venoms have been used as traditional Chinese medicines for hundreds of years, and they have a positive effect on autoimmune inflammatory diseases such as rheumatoid arthritis. However, their active, effective ingredients and their mechanisms of action are not fully understood yet. Studies have shown that snake venom is a mixture of proteins and peptides with various biological activities, including anti-tumor, anti-inflammatory, anti-stroke, and analgesic activities [1]. For example, snake venom extracts such as Cathelicidin-BF and Cathelicidin-CATH can inhibit pro-inflammatory cytokines which offer indicating effective anti-inflammatory activity [2,3,4]. This also suggests that screening snake venom compounds is an effective way to discover new molecules against inflammation and other diseases. Due to the large binding area of TNFR1, small molecules may not have enough inhibitory effects [5]. Peptides are attracting increasing attention due to their specific biochemical and therapeutic features, such as diverse bio-functionalities based on their components (amino acids) and high binding affinity with specific targets in a wide range, despite the fact that small molecules still dominate the therapeutic industry [6,7]. Therefore, it can be postulated that the peptides are more suitable as TNFR1 inhibitors [8].

Phage display is a powerful tool for developing new peptide drugs, since it can largely maintain the conformations and functions of the expressed protein and peptide simultaneously, which could maximize the retention of their biological activities with little risk of the recombinant phage infecting the host [9]. Phage display technology was originally a molecular display technology used to study the interaction between proteins. The genes were expressed on the surface of phages and interacted directly with various specific targets, making it a powerful and commonly used high-throughput screening tool that can make natural product ligands quickly connect to various specific cellular targets, including enzymes and membrane receptors [10]. In the T7 phage display system, the gene encoding the target peptide is inserted into the genome of phage T7 and then transduced into *Escherichia coli* cells. The peptide sequence is then fused to the C-terminus of the 10B capsid, and, thereby, the protein is expressed on the surface of phage particles [11]. This displayed phage can then be screened against the target proteins immobilized on the surface of the ELISA plate to detect ligand–receptor interactions. In this way, large peptide libraries can be presented on the surface of the phage and panned during repeated cycles, including binding, washing, elution, and amplification. Thereafter, by sequencing the genome of the gradually enriched phage, the displayed peptide sequence can be obtained, and the peptide can be synthesized in a recombinant or synthetic form. Finally, unique binding agents with high affinity and specificity for the desired target can be identified [12]. Phage display peptide libraries usually contain up to 10^10^ diverse variants [10]; these variants can make peptides appear on the phage surface in a variety of sizes and structures. Natural peptides that are directly separated using traditional separation methods, including high-performance liquid chromatography (HPLC), are usually present in complex mixtures of biological components at relatively low concentrations. In contrast, phage display represents a more economical and effective option to select specific peptide ligands that interact with inflammatory mediators [13,14]. For instance, two peptides of Hydrostatin-SN1 [15] and Hydrostatin-TL1 [16] with anti-inflammatory activity have been screened out from the phage display peptide library generated on the toxins of the sea snake. Both peptides can specifically bind to TNFR1 and inhibit its downstream pathways, resulting in an anti-inflammatory effect. It also shows the advantages of phage display peptide library technology in screening biotoxin peptide drugs.

Tumor necrosis factor (TNF-α, TNF) is an inflammatory cytokine with multiple functions [17]. It is produced by cells of the central inflammatory cell type and serves as the primary inflammatory stimuli to mediate inflammation through interaction with the tumor necrosis factor receptor (TNFR) [18]; the receptor activation triggers important intracellular signaling pathways, including the mitogen-activated protein kinase (MAPK), nuclear factor kappa-B (NF-κB), and Janus kinase (JAK)-signal transducer and activator of transcription (STAT) pathways [19,20,21]. It is necessary for control of the inflammatory process and bacterial and viral infections, but it can also promote autoimmune diseases and cancer [22,23,24]. The biological functions of TNF-α are mediated by two different receptors, TNFR1 and TNFR2, in the cell membrane. Mechanisms that shut down the inflammatory response are of paramount importance in return to homeostasis [25]. The current research focuses on anti-inflammatory drug trends to identify new small molecules that can directly bind to TNF-α and/or TNFR1 to prevent TNF-α from interacting with TNFR1, thus modulating downstream signaling pathways [26]. However, inhibiting TNF-𝛼 occasionally has adverse effects, including life-threatening infections, such as reactivation of hepatitis B and tuberculosis [27,28]. In addition, TNF-α blockers cannot show efficacy in diseases where TNF-α acts as the disease-promoting factor, including multiple sclerosis and heart failure. This may reflect that TNF-α blockers prevent not only TNFR1 signal transduction but also the activation of TNFR2 [29,30]. Therefore, we would practice in developing alternative therapeutic interventions for TNFR1 rather than TNF-𝛼 [31,32]. In addition, anti-inflammatory drugs on the market and in research usually have significant side effects, particularly when long-term use is involved [33,34]. Consequently, the peptides that block TNFR1 have great potential in clinical drug research and development. 

Accordingly, we chose to screen the TNFR1-binding peptide from the *D. acutus* venom peptide library, which has potential value in drug applications. Therefore, we first constructed the T7 phage library of *D. acutus* venom and then conducted biopanning to obtain potential binding sequences. Afterwards, computer simulation methods were applied to analyze the three-dimensional structures of the peptide gained and predict the binding sites between the peptide and TNFR1. After the peptide was synthesized, SPR technology was used to determine whether specific affinity between the peptide and TNFR1 existed so that a peptide with potential anti-inflammatory activity could be obtained.

## 2. Results

### 2.1. Construction of the D. acutus Venom Gland T7 Phage Display Library

**Figure 1:**
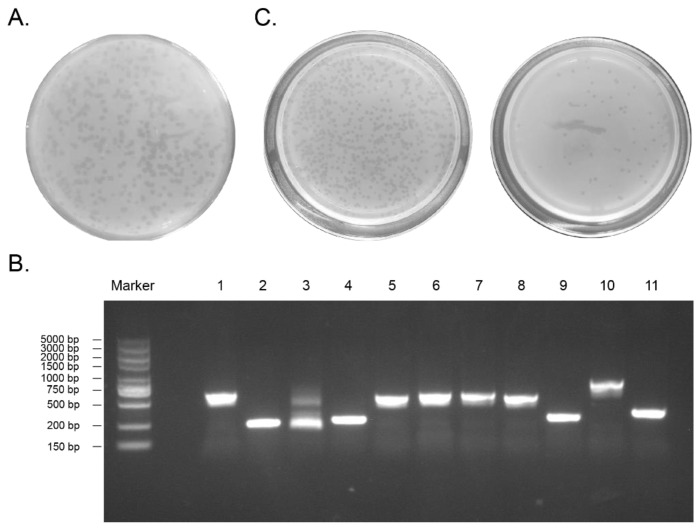
The quality controls of the library. (**A**). Each transparent spot is a plaque on which the titer of the library is counted. Based on the plaque in this plate (dilution 10^5^), we can accumulate the titer of the original library. (**B**). The gel electrophoresis image of the PCR results of the genes contained in the original library showed that fragments of various sizes were successfully inserted into phage. (**C**). The titers of the amplified library: the left is the plate diluted 10^7^ times; the right is the plate diluted 10^8^ times.

After testing, the initial titer of the library was 1.2 × 10^6^ pfu/mL (Figure 1A). Eleven plaques from the original library were randomly selected, and genes contained in the plaques were amplified by PCR. The products were analyzed by gel electrophoresis, which confirmed that gene fragments of various sizes had been successfully inserted into the phage (Figure 1B). PCR results also showed that 100% recombination was detected in the original library, indicating the high quality of the library for screening. The initial library was amplified to a titer of 7.6 × 10^10^ pfu/mL (Figure 1C), which can meet the requirements of the next biopanning experiment.

### 2.2. Obtaining Peptide DAvp-1

The ELISA plate coated with TNFR1 had an average of 2.537 of the OD 450–OD 630 value, compared to 0.018 for the uncoated control group, showing that TNFR1 has been successfully coated on the plate. This assured that the binding peptides from the phage display library to TNFR1 can be detained on the plate surface. This plate was used to conduct three rounds of screening; after selection, single colonies were randomly picked for PCR and sequencing. Through a series of biological analyses, including sequence length, solubility, and simulated affinity, the DNA sequences containing 3′-AAT TCA CTA GTC CTT CGA GGG AGG ATG AGA GAC GTA AAG GTA CGG GAT GAT GGA AGA AAA TCA CCC AGC CAC CAT AGC AAA TTT TCA GGA GGA ACA AGA AAC TGG CAA AAA CTA GTC AAG CTT-5′ were acquired. The sequence was analyzed by BLAST using BioEdit 7.0.9.0 software. The result of this blast is the sequence of *Protobothrops mucrosquamatus* solute carrier family 39 member 6 (SLC39A6), mRNA, with sequence ID: XM_015821691.1. The matching range is 2281–2336, with a matching degree of 87.9 bits (47), 53/56 (95%), and the interval is 0/56 (0%). The corresponding amino acid sequence is NSLVLRGRMRDVKVRDDGRKSPSHHSKFSGGTRNWQKLVKL and was named DAvp-1.

### 2.3. Structure of DAvp-1 and Docking Model

**Figure 2:**
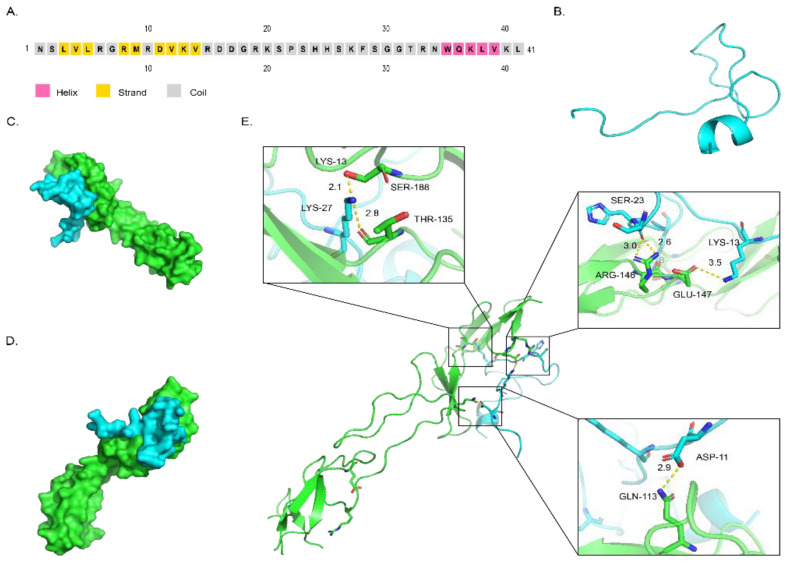
The spatial structures of DAvp-1 and its binding configurations with TNFR1. (**A**). The secondary structure of DAvp-1. (**B**). Three-dimensional diagram of DAvp-1 structure is shown in cartoon representation in cyan. (**C**,**D**). The different visual angles of the binding between DAvp-1 and TNFR1 shown as surface diagram, with DAvp-1 in cyan and TNFR1 in green. (**E**). The diagram of the hydrogen bonds between DAvp-1 and TNFR1, with the diagram of DAvp-1 and TNFR1 shown in cartoon representation, except for binding residues shown in stick representation, with DAvp-1 in cyan and TNFR1 in green. The hydrogen bonds are shown as dashed yellow lines, and the helical elements and residues participating in hydrogen bonds are labeled.

The secondary structure of the DAvp-1 peptide was predicted on the PSIPRED server. As shown in Figure 2A, the main region of DAvp-1 was composed of partial helixes and coils. Its three-dimensional structure was simulated on the SWISS-MODEL website (Figure 2B), and the template sequence number applied is 3qxb.1. A. HPEPDOCK. Server was used to predict the possible binding sites between TNFR1 and DAvp-1; Pymol 2.5.0 was used to visualize the binding between them, and the surface binding model between TNFR1 and DAvp-1 is shown in Figure 2C,D. As shown in Figure 2E, two hydrogen bonds were formed between LYS-27 of DAvp-1 and SER118 and THR-135 of TNFR1 with lengths of 2.1 Å and 2.8 Å, respectively; one hydrogen bond with a length of 2.9 Å between ASP-11 of DAvp-1 and GLN113 of TNFR1; and one hydrogen bond with a length of 2.9 Å between SER-23 of DAvp-1 and ARG-146 of TNFR1. There are two hydrogen bonds with lengths of 2.6 Å and 3.0 Å, respectively; a hydrogen bond of 3.5 Å is formed between GLU-147 and LYS-13. These connections prove they have a good mutual relationship.

### 2.4. Peptide Synthesis of DAvp-1

**Figure 3:**
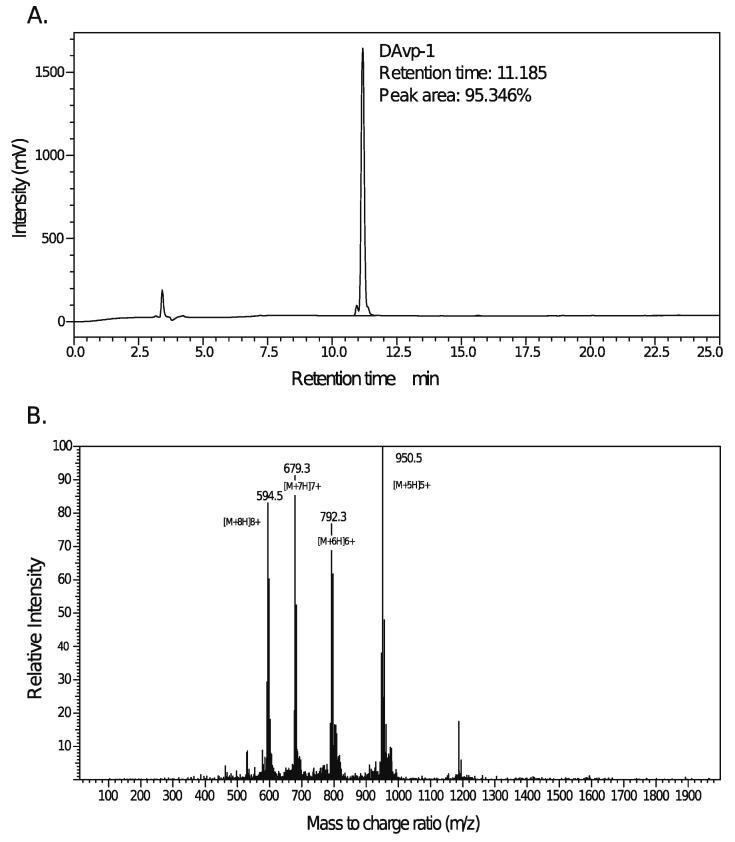
Detection and verification of synthetic DAvp-1. (**A**). The highest peak is DAvp-1 in HPLC, this showed that the purity was 95.346%. (**B**): With MS, peaks mean mass-to-charge ratio of [M+8H]^8+^, [M+7H]^7+^, [M+6H]^6+^, [M+5H]^5+^, respectively. The molecular mass of DAvp-1 (4747.5) was calculated from these charges.

DAvp-1, which appeared in the white lyophilized powder, was synthesized following the solid phase peptide synthesis (SPPS) Fmoc protocol, and Mass Spectrometer (MS) and HPLC were used for quantitative and qualitative verification. A purity of 95.346% was determined with the HPLC methodology (Figure 3A). The mass-to-charge ratios of [M+8H]^8+^, [M+7H]^7+^, [M+6H]^6+^, and [M+5H]^5+^ were 594.5, 679.3, 792.3, and 950.5, respectively, and the highest peak of [M+5H]^5+^ was selected to calculate the molecular weight (MW). The formula is

(1) $$\mathrm{MW}=\frac{m}{z}\times C-C(1)$$

where MW is the molecular weight, $\frac{m}{z}$ is the mass-to-charge ratio, and C is the charge. A molecular weight of 4.7475 kDa for DAvp-1 was obtained (Figure 3B) based on the MS result. The DAvp-1 molecule could be dissolved in the ultrapure water, 1 × phosphate buffer saline (PBS) (pH 7.1) and dimethyl sulfoxide (DMSO), respectively. A series of subsequent experiments could be conducted using DAvp-1 due to its good solubility.

### 2.5. Surface Plasmon Resonance Measurements

We investigated whether DAvp-1 targets binding to TNFR1 using surface plasmon resonance (SPR) analysis using BIAcore T200 and CM5 sensor chips. The ligand coupling level RL was calculated by the following formula:

(2) $$\mathrm{Rmax}=\frac{\mathrm{analyteMW}}{\mathrm{ligandMW}}\times \mathrm{RL}\times S$$

**Figure 4:**
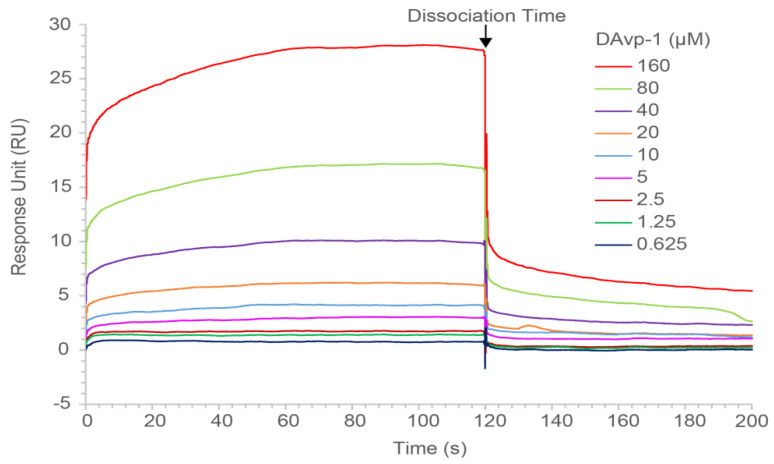
SPR sensorgram indicated the DAvp-1 applied at the indicated concentrations to the TNFR1 immobilized on the CM5 sensor chip. The lines in different colors represent different concentrations. All lines in the figure reflected the data after subtracting the blank chip control and solvent blank control, the dissociation began at 120 s.

Rmax represents the maximum binding capacity of the chip surface, while response unit (RU) is usually substituted in the protein test. The analyte MW and ligand MW indicated the molecular weights of protein TNFR1 and polypeptide DAvp-1, respectively. Sm is the stoichiometric ratio, set to 1. The calculation resulted in 597.17 RU for RL. The actual coupling amount in the experiment is 1 to 2 times over the RL, so we decided to couple 1000 RU TNFR1. The concentrations of DAvp-1 flowing through the chip from 0.625 μM to 160 μM were applied. The SPR result showed a dose-dependent resonance when DAvp-1 flowed through TNFR1 immobilized on a biosensor chip, demonstrating the direct combination of DAvp-1 with TNFR1 (Figure 4). BIA evaluation 3.2 software was used to analyze the binding curves. According to the fit of the affinity, the equilibrium dissociation constant (KD) for DAvp-1 binding on TNFR1 was found to be 45.38 μM.

## 3. Discussion

Natural peptides directly separated by traditional separation methods (such as HPLC) usually output in a complex mixture of biological components at a relatively low concentration. In contrast, phage display technology is a powerful and commonly used high-throughput screening tool [12] that can magnify components with low richness, potentially functioning pharmacologically. Therefore, the phage display technology can be used to efficiently screen the active constituents from the natural product pools, showing a more economical and effective option for selecting specific peptide ligands that interact with inflammatory mediators such as TNFR1 [14,35]. Our experiment successfully constructed a phage display library of *D. acutus* venom with premium quality and a high recombination rate. We did not find any review articles that summarize the recombination rates of the original phage library in previous studies, but a recombination rate close to 100% in our original library is extremely unusual, which provided a rich display peptide library for the subsequent screening of anti-inflammatory or other functional peptides. At the same time, this is the first *D. acutus* phage library to be constructed, and it will be used for other active ingredients screening later on.

After three rounds of screening, several sequences were obtained. It is impossible to synthesize all sequences obtained from the screening because of the high cost of peptide synthesis and the long time required. Therefore, we should try to selectively synthesize the peptides that can bind with TNFR1 and have the most potential to develop anti-inflammatory drugs. Suitable peptides were selected based on these criteria: a sequence that cannot be too long to cause difficulty in synthesis and a restriction under 45 amino acids that is mechanically stable and allows for easy modification, scaling up, etc. [8].

Homologous modeling has been developed for predicting the 3D structure of proteins and peptides originating from various organisms, and this most accurate modeling method was widely used in structural biology [36]. Software simulations were applied to test if there was a positive interaction with TNFR1 [37]. After analysis, it was believed that DAvp-1 might have a binding force with TNFR1, and therefore, it could inhibit downstream pathways and play an anti-inflammatory role [38]. DAvp-1 was selected for synthesis, and subsequent experiments were performed to verify its affinity. After synthesis, a dissolution test showed that DAvp-1 has good solubility and can be used in a series of subsequent experiments. A promising solubility is conducive to the development of drugs [39].

SPR technology was developed for measuring the dynamic interaction between ligands and receptors in a fluid environment to calculate affinity in real-time. This technique can verify the theoretical result of the simulation [40]. SPR technology was used to prove that DAvp-1 and TNFR1 have a good binding force. This verified that experiment successfully screened out the peptides that can bind to TNFR1 from the phage library of *D. acutus* venom, providing theoretical and experimental bases for the subsequent investigations of the anti-inflammatory activity and anti-inflammatory mechanism.

In conclusion, this study successfully screened out DAvp-1 from the phage library of *D. acutus* venom that could bind specifically to TNFR1, suggesting that DAvp-1 can be used to develop drugs for TNFR1-associated diseases. Additionally, our experiments emphasized that the method used in this study can screen potential pharmaceutical ingredients out from natural product pools that have been proven to have an effect in inflammation and, to a certain extent, can analyze the key ingredients and steps in compound preparation. Although we successfully screened the peptide DAvp-1, which has a binding force with TNFR1, and this peptide has the potential to be developed, its inhibitory or agonistic effect is not yet clear; thus, we will explore its anti-inflammatory mechanisms through subsequent molecular and cellular experiments as well as inflammation-induced animal models so that theoretical and applied explorations of DAvp-1 as an anti-inflammatory candidate can be prompted. In addition, we could not prove whether the conformation of DAvp-1 retains its original conformation in venom, which also needs to be verified by subsequent experiments. Nevertheless, the affinity between DAvp-1 with TNFR1 can promote the development of peptide-based delivery systems that could conjugate with non-peptidic motifs as genes or drugs [8,41]; this indicates that DAvp-1 may also be used as a drug carrier targeting TNFR1.

## 4. Materials and Methods

**Figure 5:**
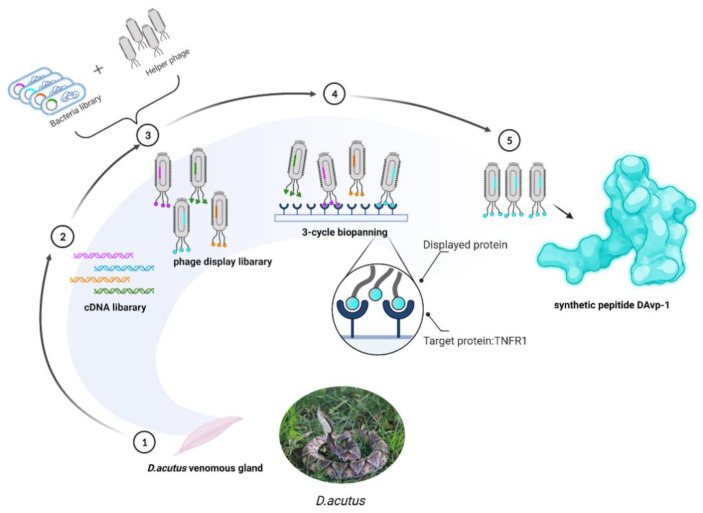
Schematic description of the established workflow for the screening of DAvp-1. (1) Extraction of venom glands from *D. acutus*. (2) mRNA was extracted from the venom glands of *D.* *acutus* and transcribed into a cDNA library. (3) Construction of a phage display library using the cDNA library of *D.* *acutus*. (4) Coating TNFR1 on the ELISA plate and performing three rounds of screening towards a *D.* *acutus*. venom phage library. (5) The screened phages were sequenced, and a series of analyses were performed to obtain DAvp-1.

A standard workflow was established for screening potential anti-inflammatory drugs that can bind to TNFR1 in *D. acutus* venom (Figure 5). First, a phage display library of *D. acutus* venom was constructed by dissecting venom glands from *D. acutus* venom. With TNFR1 as the target substrate, three rounds of screening were performed, and the selected peptide was named DAvp-1. After DAvp-1 was synthesized, the binding force between DAvp-1 and TNFR1 was verified.

### 4.1. Tissue Sampling and Construction of the cDNA Library

The snakes used in this study were *D.* *acutus* from the Huangshan Snake Park in Huangshan, China. The animals were anesthetized by pentobarbital sodium via intraperitoneal injection, and then the venom glands were dissected out, placed in liquid nitrogen for 2 h, and stored at −80 °C for later use. For total RNA extraction, the venom gland tissue was quickly transferred to a mortar pre-cooled by liquid nitrogen, and the tissue was ground until it became powder. The total RNA was extracted and purified from the tissue powder using RNA iso Plus (Takara, Shiga, Japan). We then performed gel electrophoresis immediately to verify the integrity of total RNA and used nanodrop (Thermo, Waltham, MA, USA) to determine whether the amount of extracted RNA met the experimental needs. Next, Oligotex (QIAGEN, Dusseldorf, Germany) was used to isolate Poly(A)+mRNA from total RNA. GoScript™ Reverse Transcription Mix (Promega, Madison, WI, USA) was employed to transcribe mRNA into the first strand of cDNA, and a second strand cDNA Synthesis Kit (Beyotime, Shanghai, China) was used to synthesize double-stranded cDNA, with DNA Blunting Kit (Beyotime, Shanghai, China) to fill in. The purified cDNA was stored at −20 °C until use. A DNA Purification Kit (Beyotime, Shanghai, China) was used for both purification and concentration of DNA in this experiment.

### 4.2. Construction of the T7 Phage Display Library

The *D. acutus* venom gland T7 phage display library was constructed using T7 Select Cloning Kit (Novagen, Madison, WI, USA) and according to the T7 Select System Manual. The *E. coli* strain BLT5403 was used as the host strain for the phage library. To make the cDNA and T7 vector arms possess the same sticky ends so that they can be connected, the cDNA needs to be modified by the designed oligos containing EcoR I and Hind III restriction sites named as Hind Ⅲ L, Hind Ⅲ R, ECOR IL, and ECOR IR, respectively. The oligo Hind III L sequence is GACTAGTAAGCTTGACTAGT, Hind III F sequence ACTAGTCAAGCTTACTAGTC, ECOR IL sequence GACTAGTGAATTCGACTAGT, and ECOR I F sequence ACTAGTCGAATTCACTAGTC. The oligos were synthesized in Takara. To make the oligo single-stranded into a double-stranded linker so that they could be connected to both ends of the cDNA, the oligos were annealed at 95 °C for 2 min, then we decreased by 0.1 °C every 8 s until it dropped to 25 °C; after that, they became linkers. They were digested with the monomeric restriction enzyme Hind Ⅲ (Beyotime, Shanghai, China) at 37 °C for 2 h, and then EcoR I (Beyotime, Shanghai, China) at 37 °C for 4 h. After purification, CHROMA SPIN™+TE200 Column (Takara, Shiga, Japan) was used to filter out the DNA fragments below 200 bp and connected T7 select vector arms (Novagen, Madison, WI, USA) to assemble T7 Select 10-3b plasmid. After packaging the recombinant plasmid with T7 Packaging Extracts (Novagen, Madison, WI, USA), a phage display library of *D. acutus* venom was obtained. 

To measure the titer of an initial library, 100 µL phage solution of different dilutions, 250 µL BLT5403 bacteria, and 3 ml heated and melted top agar were added into a sterile 15 mL tube without enzymes, and quickly turned upside down and shaken vigorously. The tube was then poured into LB boards, in which the temperature was kept at 37 °C. After the top agar had cooled down and solidified, the board plates were placed upside-down in a constant temperature incubator at 37 °C for 3–4 h. Each plate was counted for the fewest visible plaques. A 10-fold dilution of the plaque number was used as the titer of the phage library. Plaques were randomly selected for PCR to identify the quality of the original library. The PCR primer sequences were T7 up, GGAGCTGTCGTATTCCAGTC, and T7 down, AACCCCTCAAGACCCGTTTA. All primers used in the experiment were synthesized by Tsingke. PCR results were sequenced to find the insert sequence of the random plaque. Finally, the phage library was amplified and stored at −80 °C until use.

### 4.3. Biopanning

The biopanning procedure comprised three rounds of selection in our experiment. Briefly, TNFR1 (Genscript, Piscataway, NJ, USA) was coated on a 96-well plate overnight according to the instructions of the Peptide Coating Kit (Takara, Shiga, Japan). To verify the success of the coating, 50 µL 100-fold diluted TNFR1 antibody was added to each well of the 96-well plate, the plate was with a sealing film, and then incubated in a shaker at 300 rpm for 2 h. After discarding the remaining liquid, the plate was washed with 1×Washing Buffer and washing was repeated 6 times in total. A total of 100 µL of diluted Streptavidin-HRP was added to each well, incubated for 45 min with shaking. After the plate was washed, 100 µL chromogenic substrate tetramethylbenzidine (TMB) was added to each well, stored in the dark, and incubated at room temperature for 15 min. A total of 100 µL of stop solution was added to each well. Finally, a microplate reader was applied to measure OD 450 and OD 630, and the calculated value of OD 450–OD 630 was compared with the standard curve to determine whether the coating was successful. All reagents related to ELISA, including TNFR1 antibody, were from a Human sTNFRI/TNFRSF1A ELISA Kit (Multi Sciences, Hangzhou, China).

The 7.6 × 10^9^ phage particles were added to the TNFR1-coated 96-well plate and then incubated at 37 °C for 2 h. Two hundred microliters T7 elution buffer were added to each well and incubated for 20 min at room temperature. We collected the eluates together after the incubation and took the 250 µL eluted phage then added it to 50 ml of BLT5403 bacteria with an OD 600 of 0.5–0.6. The mixture was incubated on a shaker at 37 °C until lysis was observed. The lysate was transferred to a centrifuge tube and then centrifuged at 8000× *g* for 10 min. After the first round of panning, the lysate was stored at 4 °C until the next round. After three rounds of panning and amplification, and the phage library obtained in the last round was stored at 4 °C. The titers of the amplified phage particles were tested after elution and amplification in *E. coli* BLT5403. After three rounds of selection, single colonies were randomly picked for PCR and sequencing. The inserted sequence was analyzed by BLAST using BioEdit 7.0.9.0 software [42].

### 4.4. Structure Modeling and Molecular Docking Study

The secondary structure of DAvp-1 was predicted using the PSIPRED 4.0 server (http://bioinf.cs.ucl.ac.uk/psipred/, accessed on 4 March 2021) [43]. The SWISS-MODEl website (https://swissmodel.expasy.org/, accessed on 4 March 2021) was used to predict the 3D structure of DAvp-1, and the lowest energy was identified [36]. We employed the protein crystal structure of human TNFR1 (PDB ID: 1TNR) using the Uniprot database. The HPEPDOCK Server (http://huanglab.phys.hust.edu.cn/hpepdock/, accessed on 20 October 2021) [44] was applied to predict the possible binding sites, and Pymol 2.5.0 (Schrödinger, New York, NY, USA)was applied to visualize these structures and to analyze the interaction mode of the docking [45]. All of the above software were used with the default parameter settings.

### 4.5. Synthetic Peptide

According to the SPPS Fmoc protocol, 400 mg of modified resin was chosen to start the synthesis of the target peptide. Twenty percent pip/dimethyl formamide (DMF) solution was added into the reactor to flood the resin fully. The reactor was put on rockers for shaking for 20 min, and the solution was filtered off. DMF was added into the reactor to flood the resin fully, and, then, the reactor was put on rockers and shaken for 1 min. The solution was filtered off again. The washing procedure was conducted three times. One hundred fifty microliters of the detection reagents A and B mixture and a spot of resin were added into one test tube. Additionally, the test tube was put into 100 °C for 20 s to check if the color of the resin was changed. A color change in the resin indicated that the Fmoc groups were removed successfully. The amino acids solution, which was prepared in advance, was added to the resin. The whole procedure was recorded on the recording chart. One milliliter of the di-isopropyl carbon di-imide (DIC)/DMF solution was added into the reactor, which was put on rockers for shaking for 1 h. A total of 150 µL of the detection reagents A and B mixture and a spot of resin was added into one test tube. Additionally, the test tube was heated at 100 °C for 20 s to check for color change in the resin again. If the color of the resin did not change, the coupling of the amino acid was completed successfully. The correlated amino acid solutions were chosen to continue the coupling of the peptide until the peptide was completed. The solution containing the crude sample was injected into the machine. The fraction was collected into clean tubes according to the HPLC analysis and the level of purity required. The fraction in different tubes was confirmed and then qualified by MS and HPLC analyses. The qualified fraction solution was pooled together for freeze-drying to obtain a lyophilized powder.

### 4.6. Characterization of DAvp-1 Binding Properties by SPR

To evaluate the binding properties of DAvp-1, affinity tests were performed using a BIAcore T200 (Uppsala, Sweden). After the chip was activated using 1-ethyl-3-(3-dimethylaminopropyl) carbodiimide hydrochloride (EDC) and N -hydroxysuccinimide (NHS), according to the concentration obtained from the preliminary experiment, TNFR1 proteins dissolved in sodium acetate (pH 5.0) with 20 μg/mL were coupled to a CM5 sensor chip until 1000 RU were obtained. We blocked the excess activated carboxyl group with ethanolamine for 7 min after coupling. At the same time, a channel in the CM5 sensor chip was set as the blank control. This blank control channel also underwent activation, coupling, and blocking, but in the coupling step, the sodium acetate without TNFR1 was used as buffer.

Various concentrations of DAvp-1 were injected separately over the chip surface with a flow rate of 10 µL/min in PBS and every concentration experienced three times. HCl-Glycine buffer (pH 2.0) was used to regenerate the chip between binding cycles. The SPR signals corresponding to the reference were subtracted from those corresponding to the surfaces with immobilized proteins. The binding curves were analyzed using BIA evaluation 3.2 software (GE Healthcare, Pittsburgh, PA, USA). The CM5 chips and all reagents used in the experiment were purchased from GE Healthcare.
